# Habitat-radiomics combining multichannel 2.5D deep learning for differentiating adrenal adenomas from metastases using automatic segmentation: a multicenter study

**DOI:** 10.3389/fendo.2026.1794301

**Published:** 2026-06-12

**Authors:** Shengnan Yin, Ning Ding, Chuqi Yang, Shaocai Wang, Mengjuan Li, Yiding Ji, Tong Liu, Long Jin

**Affiliations:** 1 Department of Radiology, Suzhou Ninth Hospital Affiliated to Soochow University, Suzhou Ninth People’s Hospital, Suzhou, China; 2 Department of Traditional Chinese Medicine, Suzhou Ninth Hospital Affiliated to Soochow University, Suzhou Ninth People’s Hospital, Suzhou, China; 3 Department of Obstetrics and Gynecology, Xuzhou Central Hospital, Xuzhou, China

**Keywords:** deep learning, habitat-radiomics, lipid-poor adrenal adenoma, medical SAM, metastasis

## Abstract

**Background:**

The qualitative diagnosis of lipid-poor adrenal adenomas and metastases presents challenges, yet there is a significant difference in their treatment principles and prognosis.

**Materials and methods:**

A total of 390 patients from two hospitals were divided into training, internal validation, and external test sets. Lesion segmentation was performed automatically using the large segmentation model Medical SAM. The 2.5D deep learning model was constructed using the DenseNet-121 architecture. Habitat-radiomics employed the K-means clustering algorithm; both habitat-radiomics and conventional radiomics features were extracted from lesion regions using the PyRadiomics toolkit, with XGBoost machine learning models subsequently developed. The fusion model incorporated the 2.5D deep learning model scores, habitat-radiomics features, and clinical features.

**Results:**

The fusion model demonstrated the best overall performance, achieving areas under the ROC curve (AUC) of 0.983, 0.913, and 0.886 in the training, internal validation, and external test sets, respectively. The standalone 2.5D deep learning and habitat-radiomics models also showed good predictive performance, with AUCs ranging from 0.847-0.980, 0.805-0.967, respectively.

**Conclusion:**

The fusion model holds potential for noninvasively differentiating lipid-poor adrenal adenomas from metastases and may provide a valuable decision-making basis for subsequent precision treatment.

## Introduction

Adrenal incidentaloma (AI) refers to an adrenal mass with a maximum diameter ≥1 cm that is incidentally discovered during imaging examinations performed for non-adrenal-related conditions (1). With the widespread clinical application of imaging equipment such as CT and MRI, the detection rate of adrenal incidentalomas has increased to 4%-7% (2). Among their pathological subtypes, benign adenomas and malignant metastases are the most common (3). The diagnosis of adrenal incidentalomas primarily relies on imaging features: an unenhanced CT value ≤ 10 HU suggests a lipid-rich benign adenoma, while characteristics such as rapid contrast washout and signal drop on MRI chemical shift imaging also aid in diagnosis (4). However, approximately 30% of adrenal adenomas are lipid-poor, which are difficult to differentiate from adrenal metastases based on imaging alone. Furthermore, data indicate that among patients with a history of primary malignant tumors, only 26-36% of adrenal masses are metastatic (5, 6). Therefore, accurate characterization remains challenging even when clinical history is considered. Given that the treatment principles and prognosis for patients with adrenal adenomas versus metastases are vastly different, it is crucial in clinical practice to fully utilize clinical and imaging information to achieve an accurate qualitative diagnosis.

With the advent of the era of large models, automatic segmentation technology is undergoing a transition from “specialized small models” to “general-purpose large models” (7). The Medical SAM model enables precise and automatic localization of organs and lesions within the medical field (8). Concurrently, advancements in AI technologies have mitigated inherent limitations in medical imaging, thereby significantly reducing physicians’ workload and enhancing lesion detection rates (9, 10).

2.5D deep learning strikes a balance between 2D and 3D CNN through “multi-channel 2D convolution, ” achieving an optimal trade-off among computational efficiency, data requirements, hardware-friendliness, and spatial information preservation (11, 12). It approximates the spatial representation capability of 3D at the computational cost of 2D, making it particularly suitable for medical imaging tasks (13). This study employs three orthogonal planes—sagittal, coronal, and axial—as parallel inputs.

Radiomics is a rapidly evolving quantitative medical image analysis technology. Its core concept involves using computer algorithms to extract a high volume of features from medical images such as CT, MRI, and PET, revealing disease information imperceptible to the naked eye and thus assisting in clinical decision-making (14–16).

Habitat-radiomics refers to the division of a study object into several “sub-regions” based on functional or environmental differences to reveal spatial heterogeneity (17). Compared to traditional radiomics, habitat-radiomics emphasizes the heterogeneity between sub-regions, thereby compensating for the information loss inherent in the assumption of homogeneity across the entire region of interest (ROI) (18). By integrating with the habitat-radiomics framework, it retains the advantage of high-throughput feature extraction while enhancing the interpretability of the biological implications of tumor heterogeneity (19–21).

Our work parallelly compares the diagnostic value of four technical approaches—2.5D deep learning, traditional radiomics, habitat-radiomics, and a habitat-radiomics-2.5D deep learning fusion model—in differentiating adrenal adenomas from metastases. It aims to maximize model performance while preserving clinical interpretability and deployment feasibility under limited sample conditions. This comparative study seeks to identify the most effective method for distinguishing adrenal adenomas from metastases and provides a new methodological framework for the precise, noninvasive diagnosis of adrenal incidentalomas.

## Materials and methods

### Patients

This study was conducted retrospectively in accordance with the ethical principles of the 1975 Declaration of Helsinki. It was approved by the Ethics Committee of our Hospital.

A retrospective analysis was performed on the clinical data of 390 patients who underwent clinically indicated contrast-enhanced CT (CECT) examinations at our hospital and Suzhou Municipal Hospital between June 2020 and March 2025 and were found to have adrenal masses measuring 10-40 mm. The enrolled adrenal lesions included 216 adenomas and 174 metastases. The diagnostic criteria for adrenal adenomas were as follows: (1) imaging and clinical diagnosis of adenoma without a clear history of malignancy; (2) regular morphology and smooth margins of the adrenal lesion; and (3) no significant change in adrenal nodule size during follow-up of ≥6 months without interventional treatment. The diagnostic criteria for adrenal metastases were: (1) a clear history of malignancy with imaging and clinical diagnosis of adrenal metastasis; and (2) imaging evidence of treatment-related changes in lesion size within 6 months during follow-up. Exclusion criteria included: (1) adrenal mass diameter <10 mm or >40 mm; (2) incomplete reference standard data or incomplete CECT protocol; and (3) follow-up duration <6 months.

**Figure 1:**
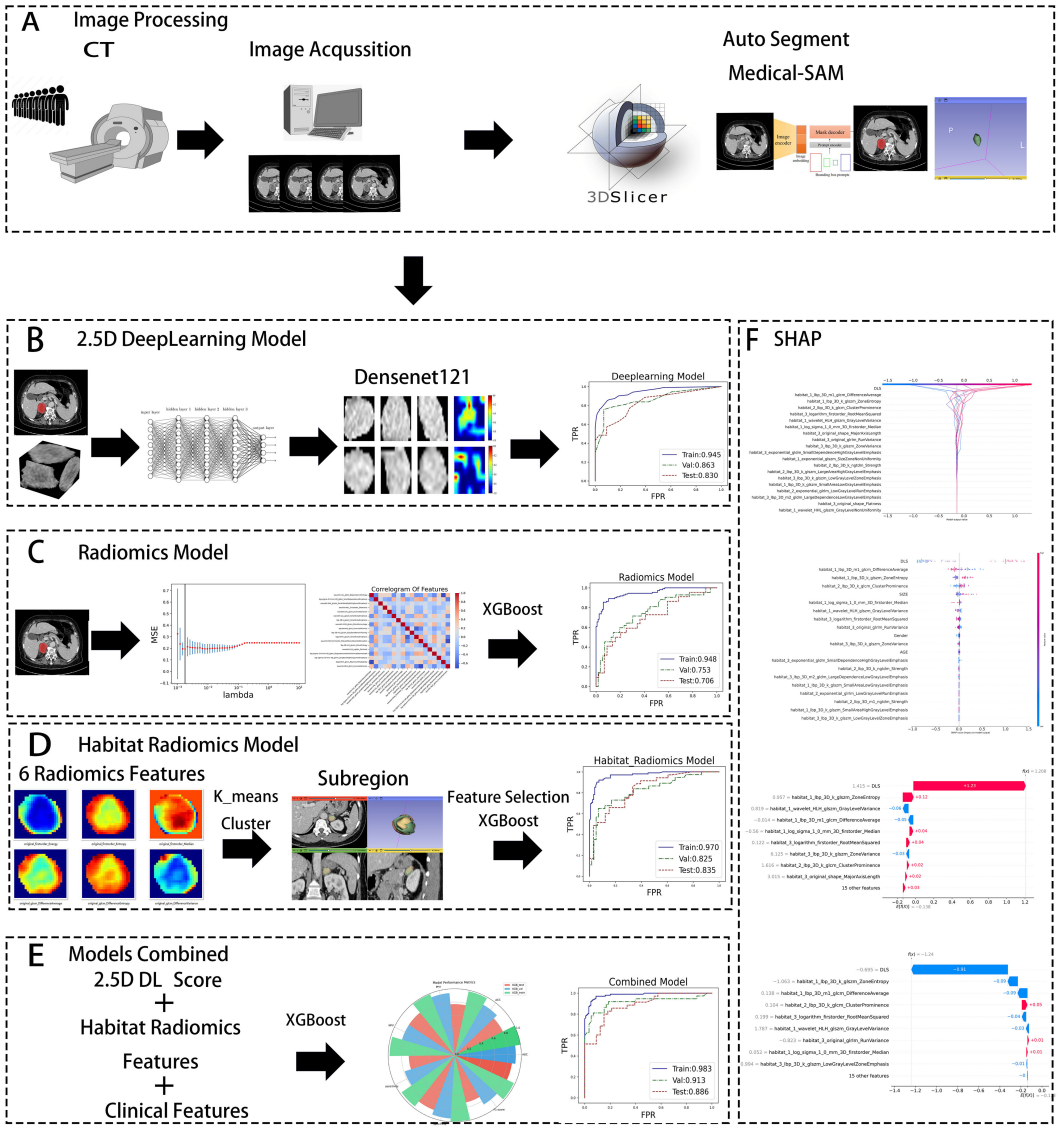
Workflow diagram for the construction of the predictive models.

Adrenal lesions selected based on the above inclusion and exclusion criteria served as the study subjects The flowchart of this study is shown in **Figure 1**.

### Protocol for abdominal CT

Abdominal CT was performed using a SOMATOM Definition Flash (Siemens, Germany) second-generation dual-source MDCT system. Scanning parameters included 120 kVp tube voltage, 300 mAs reference tube current, 5 mm slice thickness and interval, and an image reconstruction thickness and interval of 1 mm.

Patients were instructed to fast for at least four hours and received pre-scan inspiratory training. After obtaining a localizer image and performing a non-contrast scan, 80 mL of ioversol contrast (HENGRUI MEDICAL HR, China) was injected intravenously at 3.5 mL/s via a high-pressure injector (ulrich GmbH & Co.KG), followed by a 20 mL saline flush. Triphasic enhanced imaging (arterial, portal venous, and delayed phases) was triggered at 28–32 s, 55–65 s, and 3–4 min after contrast administration, respectively. The arterial phase was accurately timed using a bolus-tracking technique.

### Automatic segmentation

The portal-phase contrast-enhanced CT images in DICOM format were exported from the Picture Archiving and Communication System (PACS) to a portable hard drive. To enhance the reliability of the analysis, the original data underwent pre-processing through resampling to a resolution of 1×1×1 mm³ before image segmentation. Subsequently, input data were standardized. The CT image data were then imported into 3D-Slicer software (version 5.6.2, https://www.slicer.org), and the automatic segmentation plugin Med-SAM Lite was used.The segmentation process was semi−automatic. A radiologist with ten years of experience used a rectangular bounding box to select the lesion and appropriately adjusted the box size to fully encompass it. The segmentation result was then computed automatically by the large model. The automatic segmentation result was reviewed by a senior attending physician and manually adjusted if necessary before being saved.

### Development of a multi-channel 2.5D deep learning model

This study utilized the Python programming language, employing PyTorch (version 1.13.1) as the deep learning framework and DenseNet-121 as the deep learning model for image processing. The DenseNet-121 network was initialized with ImageNet pretrained weights. Subsequently, the images were cropped and resized to a uniform dimension of 256 x 256 pixels using linear interpolation. The resolution of the image dataset was adjusted to 224×224 pixels using the Transform library from Torchvision (version 0.14.1), and the pixel values were normalized to a fixed range (0, 1). The coronal, sagittal, and axial slices showing the largest cross-section of the lesion (224×224×3 voxels) served as the input dataset for constructing the 2.5D DL model. Data augmentation (including random rotation, flipping, scaling, and contrast adjustment) was applied to the training set. Hyperparameters were tuned via 5-fold cross-validation on the training set using grid search. During training, the network parameters were iteratively updated through backpropagation guided by the cross-entropy loss function. The model employed the Adam optimizer and was trained for 500 epochs. The learning rate was initialized at 0.01, the batch size was set to 64, and L2 regularization along with an early stopping strategy were applied to prevent overfitting. The 2.5D DCNN model generated a probability score for each case, indicating the likelihood of the lesion being an adenoma or a metastasis.

### Radiomics, habitat-radiomics feature extraction and model construction

Following the guidelines of the Image Biomarker Standardization Initiative (IBSI), radiomic features were extracted using the PyRadiomics toolkit (https://pyradiomics.readthedocs.io) (22). First, conventional radiomics analysis was performed: after initial feature extraction, discriminative features were selected sequentially through Mutual Information (MI), Minimum Redundancy Maximum Relevance (MRMR), LASSO regression, and Pearson correlation analysis, resulting in 31 features for model construction with XGBoost.

In parallel, habitat-radiomics analysis was conducted: local radiomic features were computed per voxel using a 1×1×3 moving window, generating six feature vectors per voxel. K-means clustering was applied to these vectors (23) to partition subregions; the optimal cluster number (K = 3) was determined by evaluating clusters from 3 to 8 using the Calinski-Harabasz (CH) index (24). Radiomic features were then extracted from each subregion with intensity normalization to 64 gray levels. Following extraction, a four-step feature selection and dimensionality reduction process was applied, yielding 24 features; further refinement via Pearson correlation (threshold >0.8) resulted in 23 final features. The habitat model was similarly constructed using XGBoost, and its performance was evaluated via ROC curve analysis, including AUC, accuracy, sensitivity, specificity, and F1-score.

### Fusion model construction and model comparison

Feature-level fusion was achieved by concatenating multimodal features into a unified feature vector. The habitat-radiomics features of adrenal lesions were extracted using PyRadiomics, followed by feature selection via MI, MRMR, Lasso, and Pearson correlation analysis. The 2.5D deep learning score was derived as the probability of malignancy output by the previously described DenseNet121 deep learning model. Significant clinical predictors (P<0.05) from the training cohort, including patient gender, age, and lesion size, were incorporated based on multivariable regression results. Subsequently, a fusion model based on these multivariate features was constructed using the XGBoost algorithm.

The diagnostic performance of the radiomics model, 2.5D deep learning model, habitat-radiomics model, and fusion model was evaluated and compared through internal validation and external testing. The SHAP method was employed to interpret the influence of individual features on prediction outcomes, thereby evaluating model interpretability.

### Statistical analysis

The statistical analysis for this study was conducted using Python 3.9 (https://www.python.org). The normality of variable distributions was assessed using the Shapiro-Wilk test. Normally distributed variables are presented as mean ± standard deviation, while non-normally distributed variables are expressed as median (Q1, Q3). Differences between groups were compared using the Mann-Whitney U test. Predictive performance was evaluated by the Area Under the ROC Curve (AUC). The LASSO algorithm and ROC curve plotting were implemented with the “sklearn” package, and t-tests were performed using the “scipy” package. A P-value less than 0.05 was considered statistically significant.

## Results

### Patient baseline characteristics and contrast clearance rate

This study enrolled a total of 390 patients: 216 in the adenoma group, aged 26–90 years; and 174 in the metastasis group, aged 35–89 years. Our hospital patients were randomly divided into a training set (226 cases) and an internal validation set (96 cases), patients from other hospitals were used as an external test set (68 cases). Among 390 cases, manual correction was required in 110 (28.2%). Statistically significant differences were observed between the two groups in terms of gender, age, and lesion size (P < 0.001). See **Table 1** for details.

**Table 1:** Patient characteristics.

Variables total	(n = 390)	Adenomas (n = 216)	Metastases (n = 174)	P	Statistic
group, n (%)				0.253	2.751
train	226 (57.9)	124 (57.4)	102 (58.6)		
val	96 (24.6)	59 (27.3)	37 (21.3)		
test	68 (17.4)	33 (15.3)	35 (20.1)		
AGE, Median (Q1, Q3)	65 (57, 71)	60 (52, 69)	68 (60, 73)	<0.001	12696.5
Gender, n (%)				<0.001	32.76
female	135 (34.6)	102 (47.2)	33 (19)		
male	255 (65.4)	114 (52.8)	141 (81)		
SIZE, Median (Q1,Q3)	20 (16.2, 25.4)	19.6(16.6, 23.2)	21.2(15.7, 30.3)	0.014	16067

### Deep learning visualization, sub-region clustering, and feature selection

**Figure 2:**
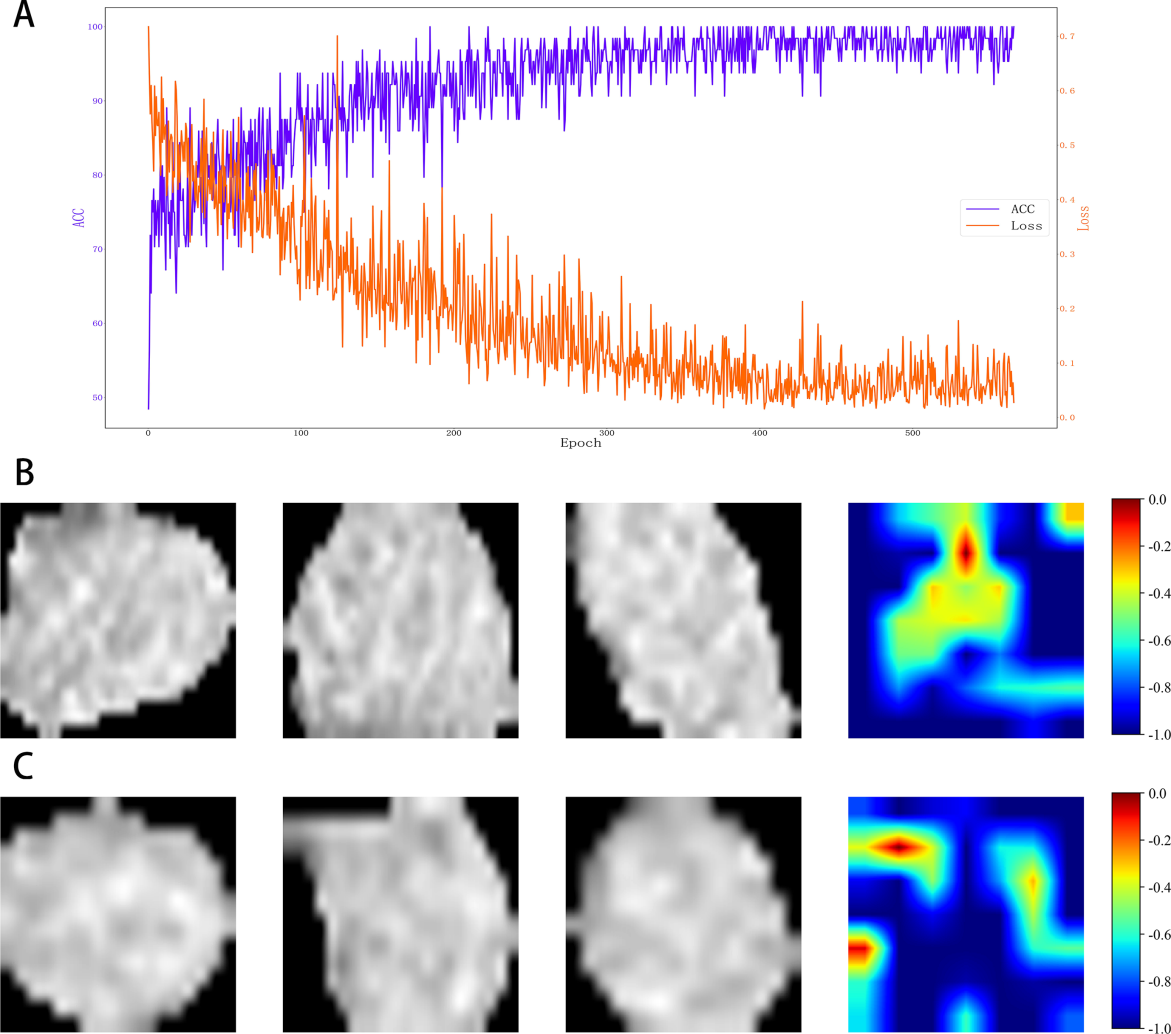
Visualization of prediction-relevant regions based on the 2.5D deep learning grad-CAM model. **(A)** Schematic diagram of the deep learning ACC/Loss curves, intuitively reflecting the training process. **(B)** Visualization of the adrenal adenoma lesion region. **(C)** Visualization of the adrenal metastasis lesion region.

The 2.5D deep learning training process based on portal-phase CT enhancement images of patients with adrenal incidentalomas can be intuitively understood from the ACC/Loss curve schematic (**Figure 2A**). The heatmaps generated by 2.5D deep learning Grad-CAM can display which regions of the image the model focuses on during prediction. **Figure 2B** illustrates the visualized regions of adrenal adenoma lesions, while **Figure 2C** illustrates the visualized regions of adrenal metastasis lesions.

For the radiomics model, a total of 1, 834 radiomic features were extracted from the portal-phase CT enhancement images of the patients. After a four-step screening process, 31 features were ultimately selected for model building.

**Figure 3:**
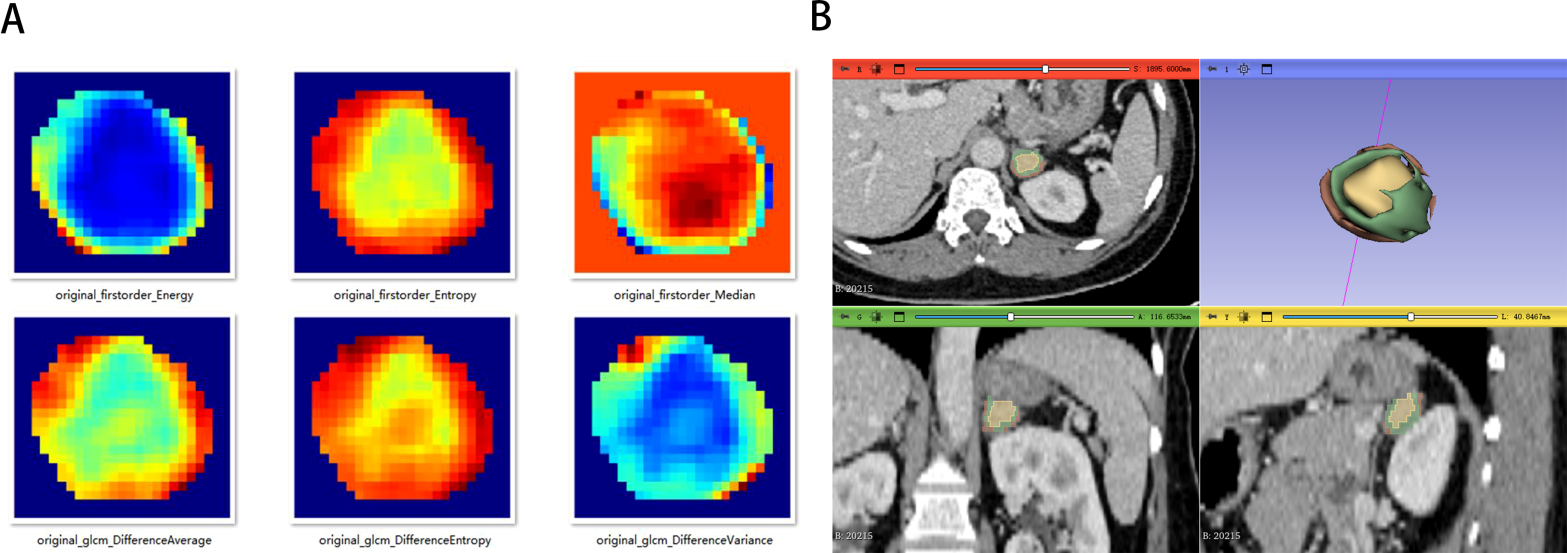
**(A) **shows a heatmap of six voxel-level radiomic features from a representative case, including three first-order features and three gray-level co-occurrence matrix (GLCM) features. **(B)** illustrates the habitat radiomics-based subregion segmentation of the lesion on portal-phase contrast-enhanced CT.

Based on the portal-phase CT enhancement images, six radiomic features were extracted from each voxel of the lesions (**Figure 3A**). After constructing the feature vector matrix, K-means clustering was performed. The optimal number of clusters was determined to be three based on the CH index, resulting in the segmentation of lesion sub-regions (**Figure 3B**). For the habitat-radiomics model, a total of 5, 502 radiomic features were extracted from the different sub-regions delineated within the lesions on the portal-phase CT images of the patients. Following the four-step feature selection process, 23 features were ultimately selected for model construction.

### Model construction and performance comparison

**Figure 4:**
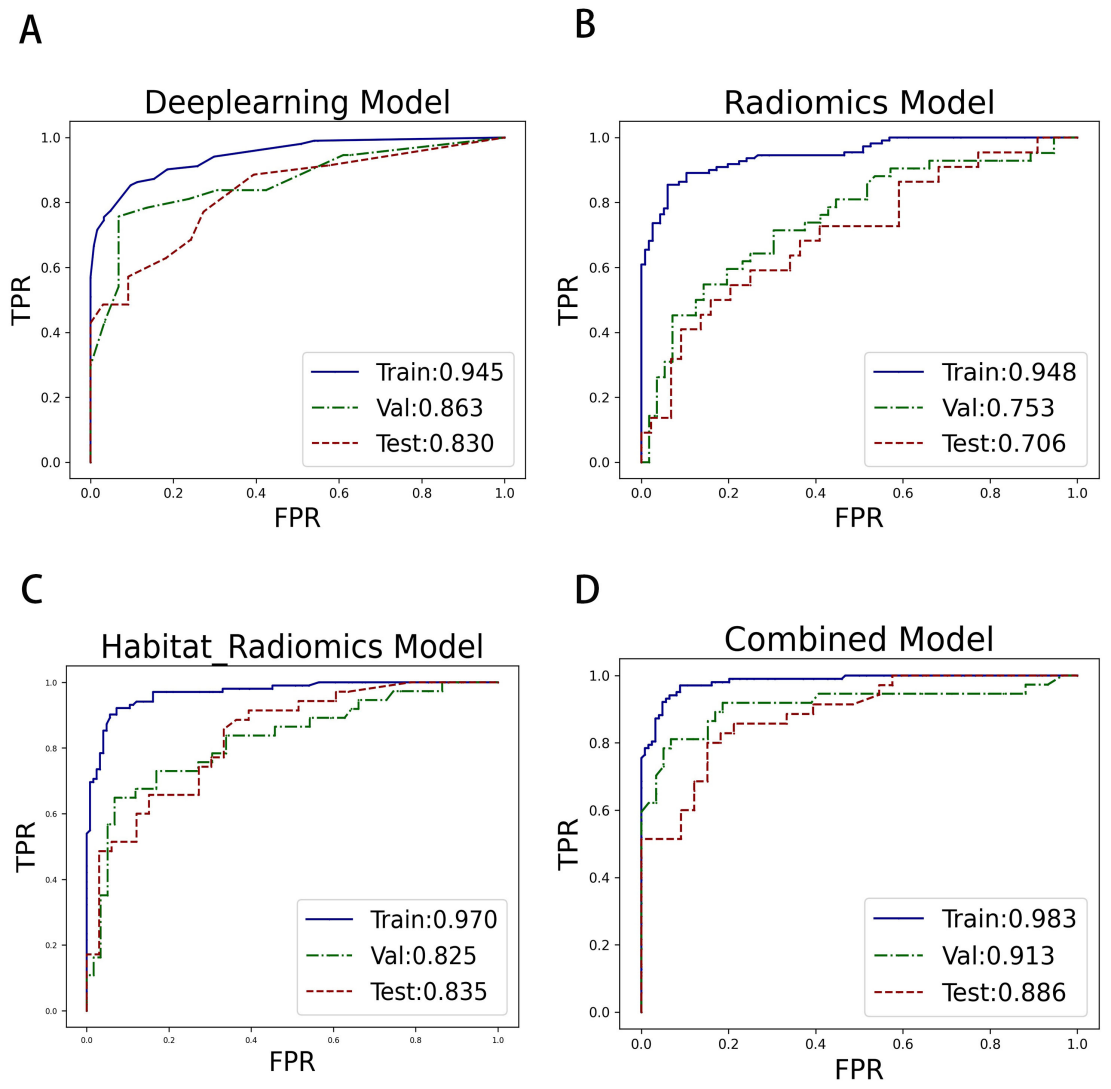
Receiver operating characteristic (ROC) curves for predicting the nature of adrenal adenoma and metastasis lesions. **(A)** ROC curve of the deep learning model; **(B)** ROC curve of the radiomics model; **(C)** ROC curve of the habitat-radiomics model; **(D)** ROC curve of the fusion model.

The 2.5D deep learning model, radiomics model, habitat-radiomics model, and fusion model were all constructed using the XGBoost machine learning algorithm. Diagnostic performance was evaluated and compared through internal validation and external testing. **Figure 4** presents the ROC curves for the 2.5D deep learning model, radiomics model, habitat-radiomics model, and fusion model. **Table 2** summarizes the ROC curve-related metrics for each model, which provide a comprehensive assessment of the diagnostic performance of the constructed models.

**Table 2:** AUC values of four models in predicting the nature of adrenal lesions.

Models	Group	AUC (95% CI)	ACC	PPV	NPV	Sensitivity	Specificity	F1-score
Deep learning	XGB-test	0.830 (0.732–0.928)	0.735	0.87	0.909	0.571	0.667	0.69
	XGB-val	0.863 (0.781–0.945)	0.865	0.875	0.932	0.757	0.859	0.812
	XGB-train	0.945 (0.913–0.977)	0.872	0.929	0.952	0.775	0.837	0.845
Radiomics	XGB-test	0.706 (0.566–0.845)	0.652	0.481	0.682	0.591	0.769	0.531
	XGB-val	0.753 (0.653–0.853)	0.714	0.694	0.804	0.595	0.726	0.641
	XGB-train	0.948 (0.917–0.978)	0.858	0.933	0.948	0.764	0.809	0.84
Habitat	XGB-test	0.835 (0.739–0.932)	0.808	0.769	0.878	0.69	0.827	0.727
	XGB-val	0.825 (0.733–0.916)	0.872	0.938	0.95	0.789	0.826	0.857
	XGB-train	0.970 (0.946–0.994)	0.983	0.99	0.992	0.972	0.977	0.981
combined	XGB-test	0.886 (0.805–0.967)	0.765	0.828	0.848	0.686	0.718	0.75
	XGB-val	0.913 (0.847–0.980)	0.865	0.833	0.898	0.811	0.883	0.822
	XGB-train	0.983 (0.965–1.000)	0.92	0.938	0.952	0.882	0.908	0.909

The results show that the diagnostic performance of the 2.5D deep learning model, the habitat-radiomics model, and their fusion model significantly outperformed that of the radiomics model, showing excellent predictive capability for distinguishing adrenal adenomas from metastases. Among them, the fusion model exhibited the best performance in both internal validation and external testing.

**Figure 5:**
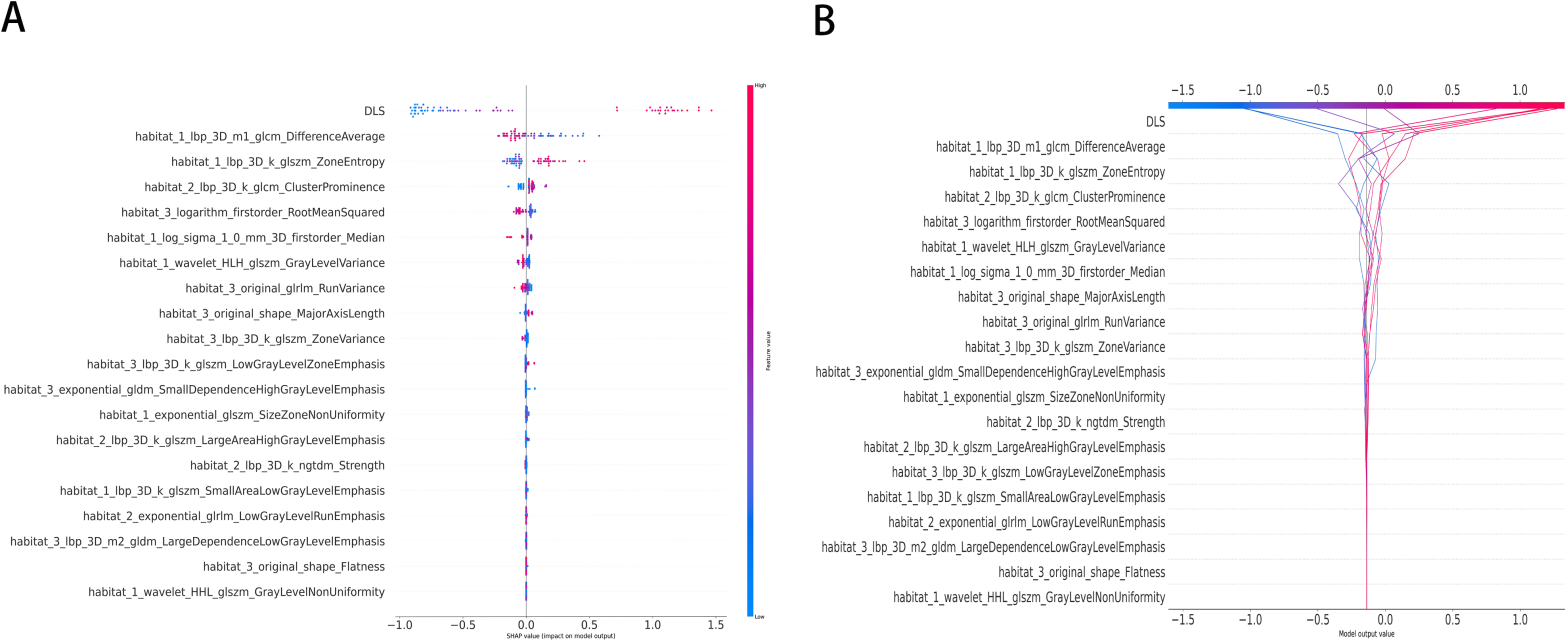
SHAP summary plot and decision plot of feature importance for the fusion model. **(A)** The dot plot illustrates the direction of contribution for each value of each variable. A larger absolute SHAP value indicates a greater influence on the predictive model. Red data points represent higher values of the feature, while blue points represent lower values. **(B)** Each line represents an individual observation sample, showing the path from the baseline prediction value to the final prediction value for that sample.

**Figure 6:**
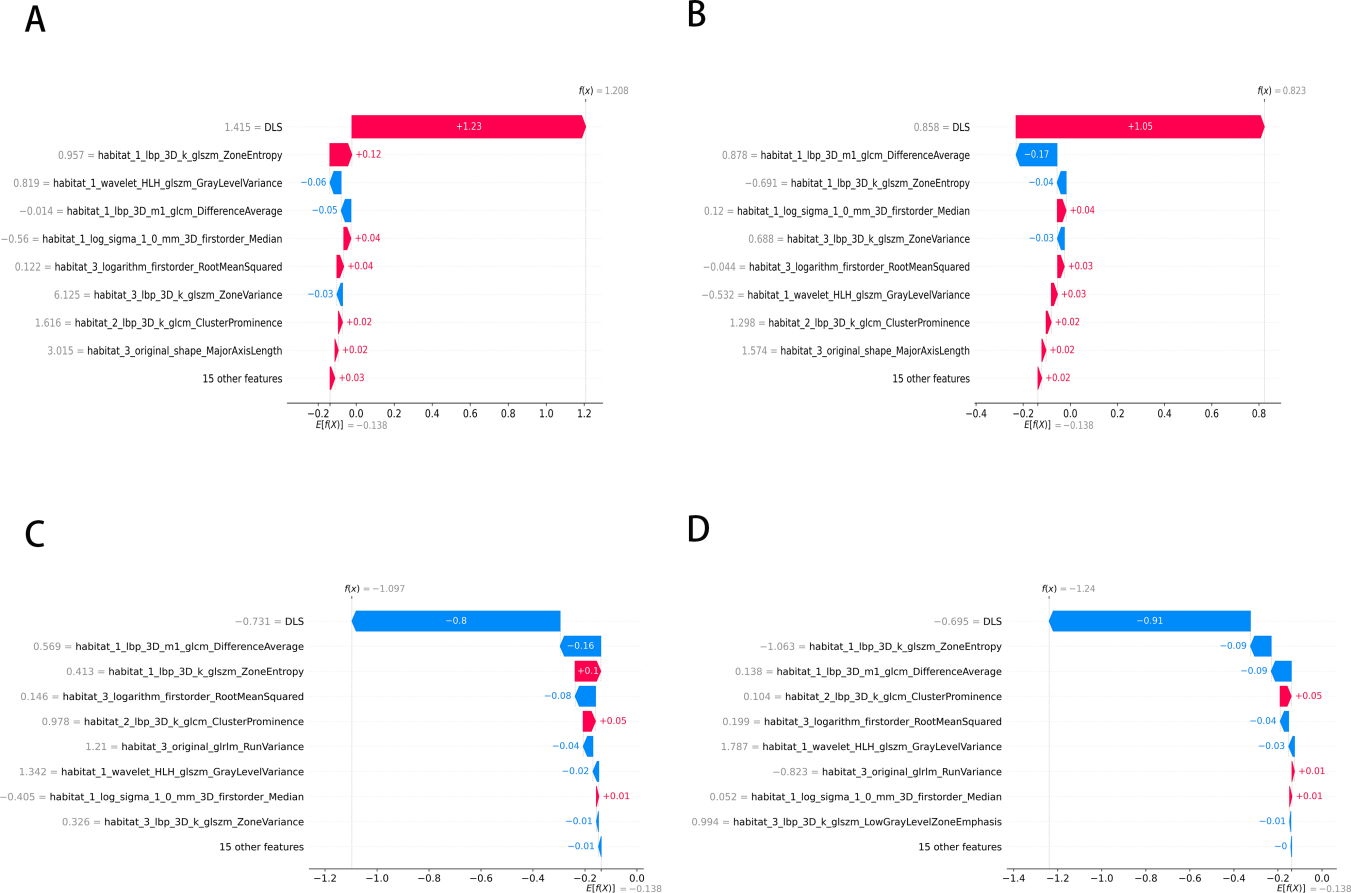
SHAP waterfall plots for individual sample predictions from the fusion model. **(A, B)** show the SHAP values for a single positive case sample (adrenal metastasis). **(C, D)** show the SHAP values for a single negative case sample (adrenal adenoma).

The SHAP summary plot (**Figure 5A**) illustrates the distribution of SHAP values for each feature across samples in the fusion model. A larger absolute SHAP value for a feature, indicated by a wider distribution range, signifies a greater impact of that feature on the predictive model. **Figure 5B** is a SHAP decision plot, where each line represents an individual observation, showing the sample’s prediction path from the baseline value to the final output. A line shifting to the right at a specific feature indicates a positive contribution of that feature to the prediction, while a shift to the left indicates a negative contribution. SHAP waterfall plots were used to provide local interpretability for individual sample predictions. **Figures 6A, B** displays the SHAP values for a single adrenal metastasis sample, and **Figures 6C, D** shows them for a single adrenal adenoma sample, visually presenting the impact of each feature on the prediction outcome.

## Discussion

Adrenal incidentalomas are common asymptomatic lesions detected in clinical imaging, including benign and malignant lesions originating from the adrenal cortex or medulla, as well as masses of extra-adrenal origin (25). Among these, 80%–85% are adrenal cortical adenomas or bilateral macronodular adrenal cortical hyperplasia, which are benign masses. Among malignant masses, adrenal cortical carcinomas account for 0.4%–4%, while adrenal metastases account for 3%–7% (26). As the two most common types of adrenal benign and malignant tumors, adrenal adenomas and metastases have significantly different prognoses. Therefore, using clinical and imaging examinations to differentiate between them and providing timely and appropriate management are crucial (27, 28). The 2023 Clinical Practice Guideline for the Management of Adrenal Incidentalomas for the Study of Adrenal Tumors states that the diagnostic value of measuring CT values on non-contrast CT has been established, but approximately one-third of adrenal incidentalomas still have HU values >10. Thus, there is an urgent need for extensive research in patients with adrenal lesions with HU >10 in distinguishing benign from malignant lesions. New technologies such as artificial intelligence and radiomics can play an important role in this process (26). Based on portal-phase contrast-enhanced CT images and employing automated segmentation techniques, this study constructed and compared in parallel a 2.5D deep learning model, a traditional radiomics model, a habitat-radiomics model, and an fusion model combining “habitat-radiomics + 2.5D deep learning + clinical features” for predicting the nature of lipid-poor adrenal adenomas and metastases. The results ultimately determined that the imaging features of the fusion model exhibited the strongest predictive power.

The automatic segmentation technology is implemented through 3D Slicer and the large segmentation model Medical SAM. It enables accurate and efficient extraction of adrenal lesion regions from contrast-enhanced CT images, and reduces human error. This technology serves as a crucial foundation for advancing toward large-scale, multicenter, real-time clinical translation of radiomics (29). Jun Ma et al. noted in their research that Medical SAM is the first foundational model for medical image segmentation. Across various imaging tasks involving CT, MRI, ultrasound, and endoscopy, MedSAM’s segmentation accuracy comprehensively outperforms SAM and is comparable to or better than specialized models such as U-Net and DeepLabV3+ (7).

Compared to 2D deep learning, the 2.5D deep learning approach adopted in our study achieves superior performance without increasing annotation burden or hardware costs. Hidemasa Takao et al. mentioned in their article that the 2.5D SSD model, by incorporating inter-slice continuity information, significantly improves precision and clinical usability without compromising sensitivity, making it more suitable for CT screening scenarios for brain metastases (30). Traditional radiomics primarily analyzes the entire lesion, with limited capability to characterize the lesion’s microenvironment (31). The parallel comparison results indicate that its AUC values for the internal validation set and external testing set significantly lower than those of other models. In contrast, habitat-radiomics, as a novel approach, can identify phenotypic variations within subregions of the lesion, thereby enhancing the ability to differentiate heterogeneity (32). This data-driven, reproducible voxel clustering method can delineate subregions reflecting tumor activity, providing valuable information for clinical diagnosis and subsequent localized treatment (33). In this study, tumor subregions were divided into three habitats (optimized using the Calinski-Harabasz index). Radiomic features were extracted from each of the three subregions to construct the habitat-radiomics model. The results show that this model also achieved high AUC values for internal validation and external testing.

The combined use of habitat-radiomics, 2.5D deep learning, and the fusion of clinical features creates a multi-level complementary system, establishing a new paradigm for diagnostic models with superior performance. This approach significantly improves prediction accuracy, providing a more feasible pathway toward precise imaging-based decision-making (34, 35). The results demonstrated AUC values of 0.913 and 0.886 for internal validation and external testing, respectively, representing the most effective method for differentiating adrenal adenomas from metastases. The high efficacy of the fusion model stems from its direct quantification of the fundamental differences between adrenal adenomas and metastases across key pathophysiological dimensions, including cellularity, extent of necrosis, and structural disorganization. Lixiu Cao et al. noted in their study that combined models based on CT radiomic features performed well in preoperatively distinguishing early adrenal metastases from lipid-poor adenomas, significantly outperforming conventional imaging indicators and single-phase models. Furthermore, the combined model exhibited greater stability, highlighting the value of feature-integrated fusion models (36).

However, the field of machine learning has long faced significant challenges in model interpretability. While the XGBoost algorithm employed in this study offers excellent predictive performance, we further applied SHAP to quantify the contribution of each feature to the model’s predictions. This approach, whether through global interpretation to analyze feature importance across the entire model or local interpretation to explain predictions for individual samples, makes the model’s decision logic transparent and comprehensible (37, 38).SHAP analysis revealed that the most influential features, in descending order, were the deep learning score, LBP texture features of habitat 1 (DifferenceAverage, ZoneEntropy), cluster prominence of habitat 2, and the first-order root mean squared of habitat 3. Together, these features provide a biological basis for model interpretability and support the feasibility of radiomics as a non-;invasive biomarker.

This study also has some limitations. First, laboratory test results including hormone levels were not incorporated, which may affect the model’s accuracy to some extent. In the future, we will further refine this information to optimize the model. Second, although this study is multicenter and the sample size is reasonable, it remains limited for machine learning purposes—particularly the external validation set—and only portal-venous phase CT images were used. In the future, we plan to expand the sample size, apply more rigorous cross-validation, and incorporate additional contrast phases to leverage washout characteristics and temporal enhancement patterns, thereby further improving diagnostic accuracy and model generalizability. Third, while we employed SHAP analysis in this study to interpret the model’s predictions, the discussion on model interpretability is still relatively limited, which represents another direction for future work. Fourth, owing to feasibility and ethical constraints, this study lacked a histopathological gold standard and primarily relied on clinical and imaging follow-up for diagnosis. This may have introduced misclassification bias that could affect model performance; nevertheless, the results from the multi-center external validation support its robustness.

In summary, we developed an automated segmentation-based fusion model combining habitat-radiomics, 2.5D deep learning, and clinical features to differentiate lipid-poor adrenal adenomas from metastases on contrast-enhanced CT images. This model exhibits strong predictive power in its imaging features, establishing a new paradigm for non-invasive and precise diagnosis of adrenal incidentalomas, and holds promise for providing a reliable basis for subsequent individualized treatment strategies. The entire workflow is highly automated: segmentation is performed semi-automatically using Med−SAM Lite (manual correction rate <30%), followed by fully automated feature extraction, deep learning scoring, and XGBoost classification. On a standard workstation (NVIDIA RTX 4090), processing takes approximately 1–2 minutes per case, which is acceptable for routine clinical use. Although the model is structurally complex, it is highly automated, computationally feasible, and can be flexibly simplified for different clinical scenarios. Further validation with expanded sample sizes is still needed to confirm its clinical value.

## Data Availability

The original contributions presented in the study are included in the article/supplementary material. Further inquiries can be directed to the corresponding authors.
